# Derinat Protects Skin against Ultraviolet-B (UVB)-Induced Cellular Damage

**DOI:** 10.3390/molecules201119693

**Published:** 2015-11-12

**Authors:** Wen-Li Hsu, Jian-He Lu, Mami Noda, Ching-Ying Wu, Jia-Dai Liu, Manabu Sakakibara, Ming-Hsien Tsai, Hsin-Su Yu, Ming-Wei Lin, Yaw-Bin Huang, Shian-Jang Yan, Tohru Yoshioka

**Affiliations:** 1 The Institute of Basic Medical Sciences, College of Medicine, National Cheng Kung University, 1 University Road, Tainan 70101, Taiwan; hsuwenli0626@gmail.com; 2 Lipid Science and Aging Research Center, Kaohsiung Medical University, Kaohsiung 80708, Taiwan; mhtsai0522@gmail.com; 3 Graduate Institute of Medicine, School of Medicine, Kaohsiung Medical University, No. 100, Shih-Chuan 1st Road, Kaohsiung 80708, Taiwan; toddherpuma@yahoo.com.tw (J.-H.L.); dermachingying@yahoo.com.tw (C.-Y.W.); 4 Laboratory of Pathophysiology, Graduate School of Pharmaceutical Sciences, Kyushu University, 3-1-1 Maidashi, Higashi-ku, Fukuoka 812-8581, Japan; mami@med.kyushu-u.ac.jp (M.N.); liubeibei.35@163.com (J.-D.L.); 5 Department of Dermatology, Kaohsiung Medical University, No. 100, Shih-Chuan 1st Road, Kaohsiung 80708, Taiwan; yup.kmu@gmail.com; 6 School of High-Technology for Human Welfare, Tokai University, 410-0321 Numazu, Shizuoka, Japan; msakaki@chime.ocn.ne.jp; 7 National Health Research Institutes, Distinguished Investigator, National Environmental Health Research Center, No. 35, Keyan Road, Zhunan Town, Miaoli County 35053, Taiwan; 8 School of Pharmacy, Kaohsiung Medical University, Kaohsiung 80708, Taiwan; tanukikimo@yahoo.com.tw (M.-W.L.); yabihu@kmu.edu.tw (Y.-B.H.); 9 Center for Stem Cell Research, Kaohsiung Medical University, Kaohsiung 80708, Taiwan; 10 Department of Physiology, College of Medicine, National Cheng Kung University, 1 University Road, Tainan 70101, Taiwan

**Keywords:** Derinat, UVB, ROS, calcium, TRPCs

## Abstract

Ultraviolet-B (UVB) is one of the most cytotoxic and mutagenic stresses that contribute to skin damage and aging through increasing intracellular Ca^2+^ and reactive oxygen species (ROS). Derinat (sodium deoxyribonucleate) has been utilized as an immunomodulator for the treatment of ROS-associated diseases in clinics. However, the molecular mechanism by which Derinat protects skin cells from UVB-induced damage is poorly understood. Here, we show that Derinat significantly attenuated UVB-induced intracellular ROS production and decreased DNA damage in primary skin cells. Furthermore, Derinat reduced intracellular ROS, cyclooxygenase-2 (COX-2) expression and DNA damage in the skin of the BALB/c-nu mice exposed to UVB for seven days *in vivo*. Importantly, Derinat blocked the transient receptor potential canonical (TRPC) channels (TRPCs), as demonstrated by calcium imaging. Together, our results indicate that Derinat acts as a TRPCs blocker to reduce intracellular ROS production and DNA damage upon UVB irradiation. This mechanism provides a potential new application of Derinat for the protection against UVB-induced skin damage and aging.

## 1. Introduction

Ultraviolet radiation (UVR) is the major etiologic factor for skin aging and its associated diseases and symptoms, such as wrinkles, epidermal pigmentation, melanin production and cancers [1]. Among the three types of UVR in sunlight, ultraviolet-B (UVB, radiation 280–320 nm) is the most cytotoxic and mutagenic stress electro-magnetic wave, and contributes to skin damage and aging [2,3]. Because the energy of UVB is sufficient to generate reactive oxygen species (ROS) in living tissue, UVB causes DNA impairment and tumorigenesis in skin associated with intracellular Ca^2+^ elevation [4,5]. However, these findings have all been independently established. Currently, the molecular and cellular links between ROS generation and intracellular Ca^2+^ elevation in living cells are not well understood. Although a large number of compounds and drugs have been developed to investigate the mechanism of UVB-induced skin damage due to ROS and Ca^2+^ elevation [6,7,8,9], acceptable models to explain relationships among UVB, Ca^2+^ entry, DNA and cellular damage are lacking because models of cellular ROS signaling remain scattered in the fields of biochemistry and physiology. Due to the disconnection between *in vitro* and *in vivo* experimental results that preclude direct comparisons, it is difficult to explain the toxic effect of UVB on skin by these scattering models. In addition, the application of several types of damage-suppressing reagents has been attempted (application to medium) *in vitro* and indirectly attempted (intra-vascular injection) *in vivo*. Thus, to reconcile fundamental differences between *in vivo* and *in vitro* experimental results, we attempted to identify a suitable reagent to study the molecular mechanism by which UVB induces skin damage and aging.

Derinat (sodium deoxyribonucleate), is a popular clinical drug with a long history and use experience in Russia. It contains DNA sodium salt isolated from the soft roes of *Acipenser gueldenstaedtii* (Russian sturgeon), which is depolymerized in 0.1% sodium chloride solution to particles with the molecular weight of 270–500 KDa through the utilization of ultrasound [10]. Clinical studies have shown that Derinat has a unique immunomodulatory function that is applicable for the treatment of pathogenesis, sepsis, inflammatory conditions and ulcers [11,12,13,14,15,16]. For example, in a study of rheumatoid arthritis, Derinat was found to suppress tumor necrosis factor alpha (TNF-α) and accelerate lymphocyte blast transformation in rats [17]. Derinat was also utilized as a clinical therapeutic against various infections, inflammatory conditions and ulcers, which are correlated with ROS production [18,19,20]. However, most of the molecular mechanisms by which Derinat functions remain unclear. Thus, Derinat would be a good candidate reagent to understand the repair mechanisms of UVB-induced cellular damage *in vivo* and *in vitro*.

Here, we hypothesized that Derinat protects skin from UVB-induced damage by inhibiting intracellular Ca^2+^ elevation induced by the transient receptor potential (TRP) channels. Our study identified the underlying mechanisms by which Derinat protects the skin from the UVB-induced destruction of skin *in vitro* and *in vivo*. To this end, we utilized keratinocytes (KCs) and human dermal fibroblasts (HDF) from human foreskin and then examined the UVB-induced impairment of the skin *in vitro*. Furthermore, for the *in vivo* studies, we determined UVB-induced skin injury symptoms in nude mice. Our results revealed that Derinat significantly protected not only skin cells, but also the epidermis of nude mice from UVB-induced damage. Taken together, these results suggest that Derinat reduces skin aging by depressing intracellular Ca^2+^ elevation and may be useful to prevent/treat age-associated diseases/symptoms.

## 2. Results and Discussion

### 2.1. Derinat Protected Skin Cells from Damage Induced by UVB Irradiation

**Figure 1:**
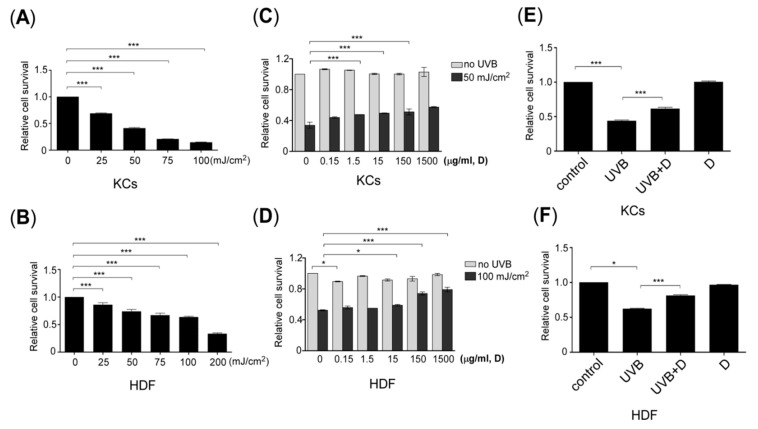
Derinat protected skin cells from UVB damage. The effect of UVB irradiation on cell viability in (**A**) keratinocytes (KCs) and (**B**) human dermal fibroblasts (HDF) (***, *p* < 0.001). The cell survival probability for cells pretreated with different concentrations of Derinat for 24 h and irradiated with or without UVB-exposure in (**C**) KCs and (**D**) HDF (*, *p* < 0.05; ***, *p* < 0.001). Further confirming the result of (**C**,**D**), in (**E**) KCs and (**F**) HDF were pretreated with 15 μg and 150 μg of Derinat and irradiated with 50 mJ/cm^2^ and 100 mJ/cm^2^ UVB, respectively. The cell survival probability was analyzed using an MTT assay after 24 h of UVB-exposure (*, *p* <0.05; ***, *p* <0.001).

To explore the effect of Derinat on UVB-induced skin damage, we first defined the effective dose of Derinat for ultraviolet-B (UVB) irradiation in skin cells *in vitro* using human keratinocytes (KCs) and human dermal fibroblasts (HDF). The skin cells were irradiated with different amounts of UVB, and the ratio of cell viability was then quantified for various radiation doses, as described in the experimental section. Figure 1A,B show that 45% of KCs and 65% of HDF survived after 50 mJ/cm^2^ and 100 mJ/cm^2^ of UVB exposure, respectively. KCs pretreated with different concentrations of Derinat for 24 h were irradiated with UVB, followed by conditioning with 15 μg/mL of Derinat for KCs (Figure 1C) and 150 μg/mL of Derinat for HDF. This approach effectively prevented UVB-induced impairment in skin cells (Figure 1D). No additional toxic effects were observed in response to Derinat treatment (Figure 1C,D). The viabilities of KCs and HDF were 70% and 78% (Figure 1E,F), respectively. These results indicated that Derinat promotes the survival ratio of KCs and HDF in response to UVB-induced damage.

### 2.2. Derinat Decreased Intracellular Ca^2+^ Elevation in Skin Cells Exposed to UVB

**Figure 2:**
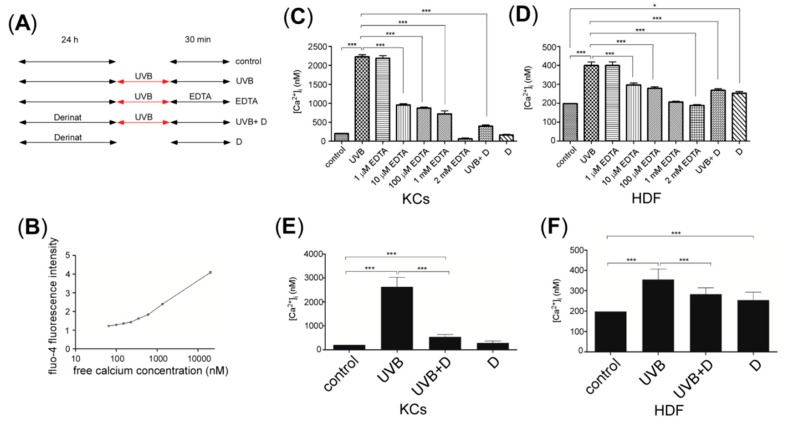
The effect of Derinat on the intracellular Ca^2+^ concentration of UVB-exposed skin cells. (**A**) Experimental design for the treatment of each group: control, UVB, EDTA, UVB+D and Derinat only; (**B**) Ca^2+^ calibration curve; (**C**,**D**), Mean value of intracellular Ca^2+^ for control, UVB-exposed, UVB-exposed with different concentrations of EDTA, UVB-exposure with Derinat and Derinat in KCs and HDF cultured in BSS for 30 min (*, *p* < 0.05; ***, *p* < 0.001); (**E**) KCs and (**F**) HDF were pretreated with or without the Derinat for 24 h and exposed to UVB. The intracellular Ca^2+^ concentration was then measured after 24 h of incubation in normal medium (***, *p* < 0.001).

Previous studies indicated that UVB increased the intracellular Ca^2+^ concentrations and contributed to skin damage and aging [21,22]. Therefore we hypothesized that Derinat protects skin cells from UVB-induced damage by blocking intracellular Ca^2+^ elevation. To test this hypothesis, we measured the intracellular Ca^2+^ concentration via fluo-4 staining. The skin cells were exposed to UVB irradiation after Derinat pretreatment for 24 h. After 30 min of UVB irradiation, the intracellular Ca^2+^ concentration was observed using an Olympus fluorescence microscope with an average fluorescence intensity of more than 150 cells. The experimental protocol for each group is presented in Figure 2A, and a Ca^2+^ calibration curve was generated to calculate the intracellular concentration in skin cells under the microscope (Figure 2B). Figure 2C,D show that Derinat almost completely blocked the UVB-induced intracellular Ca^2+^ elevation in KCs, whereas it showed a smaller but statistically significant reduction in the Ca^2+^ elevation in UVB-treated HDF. These results suggested that Derinat blocked the UVB-induced Ca^2+^ elevation in both KCs and HDF cells. We also tested whether external cellular environment drives the UVB-induced increase in the intracellular Ca^2+^ concentration. The application of Ethylenediaminetetraacetic acid (EDTA), a chelator of extracellular Ca^2+^, reduced the intracellular Ca^2+^ concentration in a dose dependent manner in skin cells 30 min after UVB exposure (Figure 2C,D). As a result, Derinat reduces the UVB-induced Ca^2+^ entry from the extracellular space into the intracellular compartment. Interestingly, Derinat also attenuated Ca^2+^ elevation in skin cells 24 h after UVB irradiation (Figure 2E,F). Perhaps Derinat blocks the initial trigger for UVB-induced Ca^2+^ elevation in skin cells.

**Figure 3:**
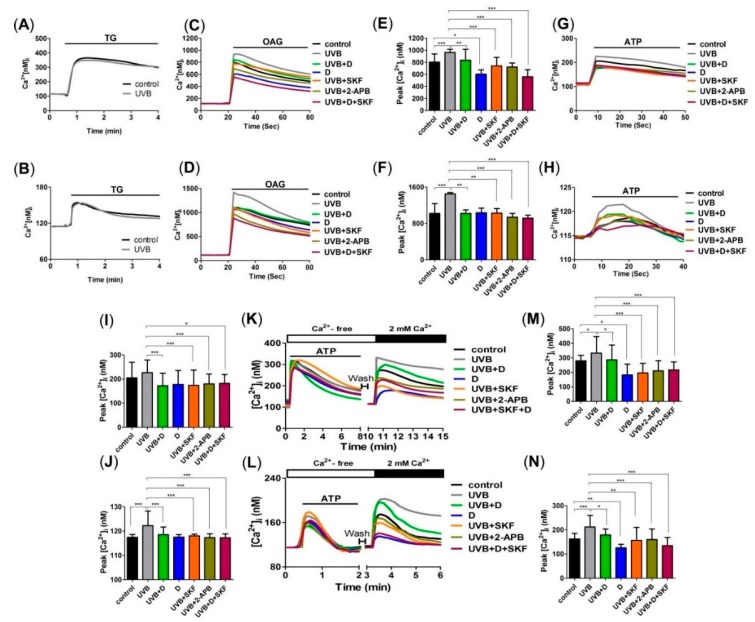
Derinat reduced UVB activated Ca^2+^ signaling in skin cells. Ca^2+^ imaging analysis of the thapsigargin (TG)-induced Ca^2+^ response after the application of 20 nM TG (small black bars) in (**A**) and KCs and 100 nM TG in (**B**) HDF (*n* = 3). After 30 min of pretreatment with Derinat (D), SKF96365 (SKF, 20 μM in KCs and 50 μM in HDF), 2-aminoethoxydiphenyl borate (2-APB, 50 μM in KCs and 100 μM in HDF) and SKF + D; OAG at 100 μM and 200 μM was applied to (**C**) KCs and (**D**) HDF, respectively, to stimulate the Ca^2+^ responses of TRPCs after 30 min of UVB exposure (*n* = 3). (**E**,**F**), quantification of the peak of the intracellular Ca^2+^ responses shown in (**C**) and (**D**) (*, *p* < 0.05; **, *p* < 0.01; ***, *p* < 0.001). Ca^2+^ imaging analysis of the adenosine triphosphate (ATP)-induced Ca^2+^ response. The application of 100 μM ATP (small black bars) in (**G**) KCs and 200 μM ATP in (**H**) HDF (*n* = 3). (**I,J**), quantification of the peak of the intracellular Ca^2+^ responses shown in (**G**,**H**) (*, *p* < 0.05; ***, *p* < 0.001). CaCl_2_ was extracellularly applied (large black bar) to increase Ca^2+^ from 0 to 2 mM and open the TRPCs after the application of ATP (small black bars) in (**K**) KCs and in (**L**) HDF in Ca^2+^-free BSS solution (open bar) (*n* = 3). (**M**,**N**) quantification of the peak of the intracellular Ca^2+^ responses shown in (**K**,**L**) after applying CaCl_2_ (*, *p* < 0.05; **, *p* < 0.01; ***, *p* < 0.001). The Ca^2+^ signals represent the mean value of 20 cells.

**Figure 4:**
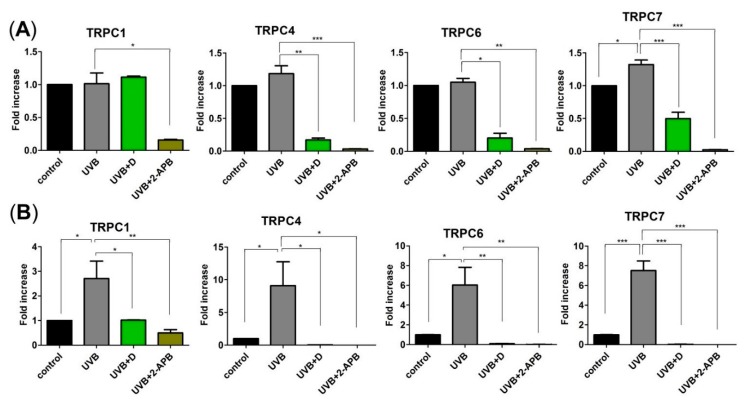
Derinat significantly inhibited UVB-induced increase of TRPC7 expression in skin cells. The cells were exposed to UVB irradiation after Derinat and 2-APB pretreatment for 30 min. After radiation of UVB for 24 h, expressions of TRPC1, TRPC4, TRPC6 and TRPC7 mRNA, which were detected from total RNA extracts of (**A**) KCs and (**B**) HDF, were measured using a qRT–PCR (*, *p* < 0.05; **, *p* < 0.01; ***, *p* < 0.001).

To investigate the mechanism by which Derinat blocked Ca^2+^ influx, skin cells were pretreated with Derinat for 30 min, and the Ca^2+^ influx activities were then detected with Ca^2+^ imaging analysis. We first used thapsigargin (TG) to raise cytosolic Ca^2+^ by depleting Ca^2+^ stores. As shown in Figure 3A,B, UVB did not affect the TG-activated Ca^2+^ influx in skin cells. We next determined whether the Transient Receptor Potential (TRP) superfamily of cation channels regulate UVB-activated Ca^2+^ entry. As diacylglycerol (DAG) activates the majority of TRP channels [23,24], and ultraviolet (UV) light irradiation increased DAG [25], a previous study hypothesized that TRP channels may contribute to UVB-induced intracellular Ca^2+^ elevation in skin [23]. We then detected whether the presence of cations precedes the activation of the TRP channels. As shown in Figure 3C,D, Derinat, 2-aminoethoxydiphenyl borate (2-APB) and SKF96365 [26,27] significantly decreased the Ca^2+^ signaling induced by a DAG analogue, membrane permeable 1-oleoyl-2-acetyl-sn-glycerol (OAG), in KCs and HDF after 30 min of UVB exposure. OAG-medicated Ca^2+^ peak was blocked by Derinat from 970 nM to 800 nM in KCs (Figure 3E) and from 1300 nM to 1100 nM in HDF (Figure 3F), respectively. According to Venkatachalam’s study published in 2007, transient receptor potential canonical (TRPC) channels (TRPCs), TRPV1 and TRPA1 are activated by DAG [24]. However, Figure 3C–F indicate TRPV1 and TRPA1 are not involved in UVB induced Ca^2+^ elevation because of no Ca^2+^ increase with 2-APB treatment [28,29]. Whereas Derinat and inhibitors efficiently blocked non-selective TRP agonist OAG-activated after 30 min of UVB exposure, we further confirmed whether TRPCs were activated by UVB. Adenosine triphosphate (ATP) was applied as a stimulator to induce TRPCs response, because ATP activates phospholipase-C (PLC) pathway, which leads to TRPCs open [30,31,32,33]. We firstly tested the effect of ATP on Ca^2+^ response in skin cells. Irradiation with UVB slightly promoted the level of ATP activated Ca^2+^ response while was reduced that minor via treating with Derinat, SKF96365 and 2-APB (Figure 3G–J). Thus, further attempt to demonstrate UVB activated TRPCs by PLC pathway stimulation. As shown in Figure 3K–N, Derinat and inhibitors restrained UVB-induced increase of Ca^2+^ influx by PLC stimulation in skin cells. Based on our results, we can’t exclude TRPM5 and TRPM7 are also involved in PLC-induced Ca^2+^ influx. Nevertheless, the population of TRPM5 in skin exists small amount [34]; TRPM7 can’t be activated by DAG. Therefore the exclusive interruption indicates Derinat reduces UVB-activated TRPCs in initiated stage. Interestingly, after 24 h of UVB irradiation, detection of TRPC1, TRPC4, TRPC6 and TRPC7, which are expressed in skin cell [34], UVB induced increase of TRPCs expression were also interrupted by Derinat and 2-APB, especially TRPC7 (Figure 4A,B). Based on the physicochemical features of Derinat and TRPCs, Derinat can block the activity of TRPCs in a similar manner as tetrodotoxin (TTX) blocks sodium channels [35]. However, unlike TTX, Derinat is negatively charged, and thus is unable to insert into a channel pore. Moreover, a heterogeneous DNA mixture would reduce UVB induced damage, because a topical solution would absorb the UVB radiation preventing cellular damage. Except against UVB-induced TRPCs activation, Derinat contains heterogeneous DNA may protect skin damage via absorbing the light under UVB range. 

Taken together, these results suggest that Derinat acts as a TRPCs blocker to reduce intracellular Ca^2+^. In the future, it is important to determine which TRPCs are involved in regulation UVB-activated Ca^2+^ elevation by the use of each TRPC siRNA.

### 2.3. Derinat Reduced Oxidative Stress Accumulation Following the UVB Irradiation of Skin Cells

**Figure 5:**
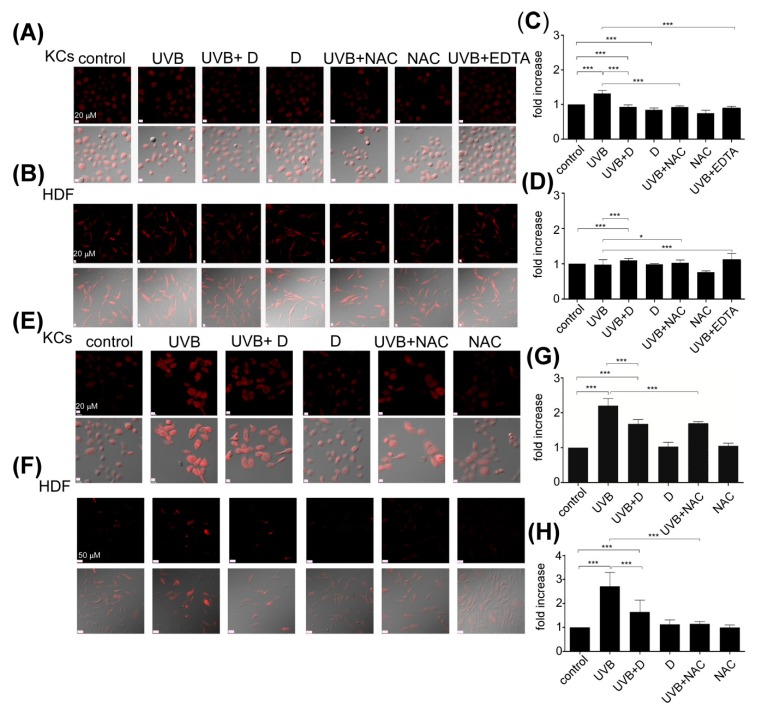
The effect of Derinat on UVB induced intracellular ROS production in skin cells. (**A**) KCs and (**B**) HDF were pretreated with Derinat and 0.5 mM NAC for 24 h and then irradiated with UVB. EDTA (1 mM) was applied after UVB exposure. The ROS generation was measured after 30 min of UVB irradiation by using 5 μM dihydroethidium (DHE) staining. The intracellular ROS production level with an average fluorescence intensity of more than 150 cells from (**A**) and (**B**) is quantified in (**C**) KCs and (**D**) HDF (***, *p* < 0.001; *, *p* < 0.01). To further investigate the intracellular ROS generation in (**E**) KCs and (**F**) HDF, the cells were cultured in normal medium for 24 h after UVB-exposure, and the intracellular ROS production was detected with a confocal microscope using DHE staining. The quantification of intracellular ROS production from (**E**,**F**) is shown in (**G**,**H**) (***, *p* <0.001).

The first response to UVB-induced intracellular ROS generation in skin cells is an elevation of intracellular c Ca^2+^ [21]. Based on our results, Derinat attenuated the UVB-induced Ca^2+^ elevation. These results suggested that Derinat protects skin cells from UVB-induced damage by decreasing the level of Ca^2+^-induced intracellular ROS production by mitochondria. We tested this hypothesis by performing the following experiments: thirty minutes after UVB exposure, the level of intracellular ROS was slightly increased by 1.25-fold in KCs (Figure 5A,C). Pretreatment with Derinat or *N*-acetylcysteine (NAC), a intracellular ROS scavenger that that reportedly inhibits UVB-induced intracellular ROS production and DNA damage in skin cells [36], suppressed the UVB-induced increased intracellular ROS levels in KCs. Incubation with EDTA after UVB exposure also produced similar results. Interestingly, the effects of Derinat and NAC on HDF did not differ after 30 min of UVB exposure (Figure 5B,D). However, both KCs and HDF exposed to UVB showed decreased levels of intracellular ROS generation after 24 h when treated with Derinat and NAC (Figure 5E,F). The intracellular ROS production levels in KCs and HDF were quantified, which showed that Derinat attenuated the UVB-induced levels of ROS production from 2.25- to 1.67-fold and from 2.52- to 1.53-fold in KCs and HDF, respectively (Figure 5G,H). Similar results were also found in the NAC pretreatment group (Figure 5G,H). Taken together, these results clearly show that the Derinat suppressed UVB-induced intracellular ROS production by reducing the intracellular Ca^2+^ elevation in skin cells.

### 2.4. Derinat Protected Skin Cells Against UVB Induced DNA Damage

**Figure 6:**
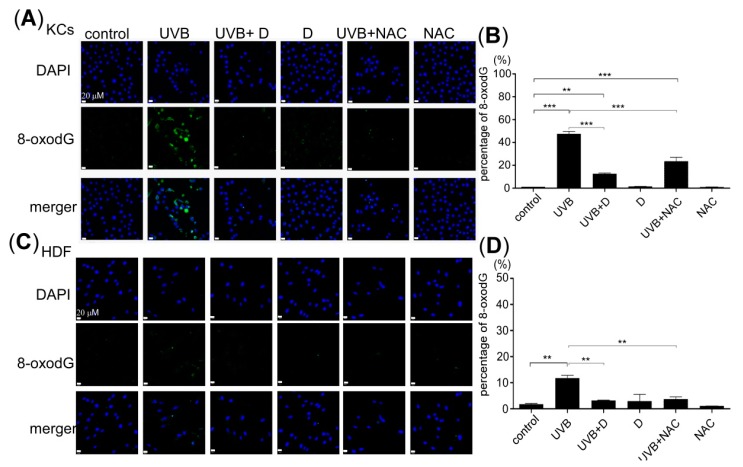
The effect of Derinat on the UVB-induced DNA damage in skin cells. 8-oxodG was detected to determine the DNA damage resulting from UVB-exposure in cells pretreated with Derinat and NAC: (**A**) KCs and (**C**) HDF. The relative values of DNA damage from 8-oxodG are quantified in (**B**,**D**) (**, *p* < 0.01; ***, *p* < 0.001). The data shown represent the average of three independent experiments.

UVB-induced intracellular ROS production via the nitric oxide pathway has already been shown to contribute to nuclear and mitochondrial DNA damage, both of which are observed when 8-oxo-2’-deoxyguanosine (8-oxodG) accumulates in skin cells [37,38]. As demonstrated in our above results, Derinat may protect skin cells from UVB-induced DNA damage by decreasing the level of intracellular ROS production. Thus, we investigated the effects of Derinat on UVB-induced DNA damage. Skin cells were pretreated with Derinat or NAC for 24 h, and the ratio of DNA damage and 8-oxodG 24 h after UVB exposure was then measured. Pretreatment with Derinat and NAC reduced the ratio of DNA damage from 51% to 12% and from 51% to 23%, respectively (Figure 6A,B). Similar results were also observed in HDF, but Derinat and NAC pretreatment reduced the ratio of DNA damage to 2% and 3%, respectively (Figure 6C,D). Taken together, these results support our above findings in skin cells: Derinat protects skin cells against UVB-induced injury by suppressing intracellular Ca^2+^ elevation, intracellular ROS production and DNA damage, which helps skin cells to avoid skin impairment and reduces the aging potential.

### 2.5. Derinat Protected BALB/c-nu Mice from UVB-Induced Skin Damage

**Figure 7:**
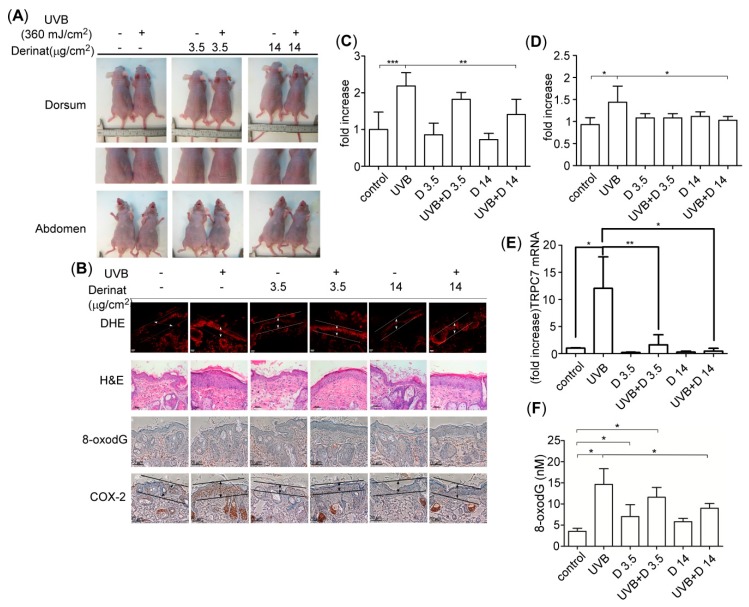
The effect of Derinat on UVB induced skin damage in BALB/c-nu mice. (**A**) BALB/c-nu mice were covered with Derinat hydrogel or pure hydrogel for 3 h and then irradiated with 360 mJ/cm^2^ UVB. After seven days, the images of animals presented UVB-induced desquamation in the dorsal areas but no effect on the abdominal areas. The middle panel shows the amplified images from the dorsal areas of UVB-induced desquamation; (**B**) Derinat protected BALB/c-nu mice skin from UVB-induced epidermal proliferation, DNA damage and cyclooxygenase (COX)-2 expression. The skins of normal and UVB irradiated mice covered with Derinat hydrogel or hydrogel were stained with DHE and H & E (hematoxylin and eosin), and the oxidative DNA damage 8-oxodG was analyzed via an immunohistochemical assay with a specific anti-8-oxodG mouse monoclonal antibody. The expression of COX-2 in the epidermis was detected with an anti-COX-2 rabbit monoclonal antibody; (**C**,**D**), the quantification of intracellular ROS production and COX-2 level from (**B**) shown in arroew indicated region includes both epidermis and dermis without sebaceous glands (*, *p* < 0.05; **, *p* < 0.01; ***, *p* < 0.001). Derinat decreased the UVB-induced level of TRPC7 (**E**) and DNA damage 8-oxodG (**F**) in BALB/c-nu mouse skin based on qRT–PCR and ELISA assay, respectively (*, *p* < 0.05; **, *p* < 0.01).

Next, we investigated whether Derinat protected animal skin from UVB-induced damage in BALB/c-nu mice by observing the desquamation, epidermal thickness, ROS production and DNA damage. Derinat is classified as a water-soluble reagent; therefore, Derinat does not easily enter skin. We cooperated with SOMAPEX BIOTECH. Co. (Kaohsiung, Taiwan) to develop a special hydrogel for Derinat (D hydrogel). Derinat hydrogel helps skin tissue absorb this drug by tuning the surface charge distribution on the pores of the skin surface [39,40]. The Derinat-containing hydrogel formed a 3-cm diameter circular chip. Three different concentrations of Derinat in hydrogels were prepared for the *in vivo* experiments: 0 (control), 3.5 μg/cm^2^ (15 μg/mL) and 14 μg/cm^2^ (60 μg/mL). During the experiment, the BALB/c-nu mice were mechanically immobilized. The skin of each mouse was covered with different concentrations of Derinat hydrogel for 3 h. After 3 h of treatment, the BALB/c-nu mice were irradiated with 360 mJ/cm^2^ UVB light [6]. After seven days, this radiation resulted in severe desquamation and erythema in the dorsal skin in BALB/c-nu mice, as indicated in Figure 7A, while 3.5 μg/cm^2^ and 14 μg/cm^2^ Derinat hydrogel mitigated this UVB-induced skin damage in animal skin. Moreover, the abdominal area of the skin of each BALB/c-nu mouse was utilized as a control, and no effect was observed in this area. Moreover, the Derinat treatment reversed the reduction of the epidermal thickness and intracellular ROS production induced by UVB (Figure 7B,C). The intracellular Ca^2+^ concentration can upregulate cyclooxygenase-2 (COX-2), which is known to be controlled by UVB induced inflammation [41,42]. The COX-2 expression was also expected to be increased in response to the UVB irradiation of the mouse epidermis [41]. Therefore, we tested whether Derinat also affects the COX-2 expression in skin cells. As shown in Figure 7B,D, Derinat treatment reduced the expression of COX-2. UVB-induced TRPC7 expression also decreased the level by Derinat (Figure 7E). Moreover, Derinat protects skin cells from UVB-induced DNA damage in BALB/c-nu mice (Figure 7B). To further elucidate the protective effect of Derinat on skin cells with UVB-induced DNA damage, we excised a small piece of dorsal skin and then extracted genomic DNA from it to quantify the percentage of DNA damage using an HT 8-oxodG ELISA kit II (Trevigen Inc., Gaithersburg, MD, USA). The quantitative analysis of 8-oxodG is displayed in Figure 7F, demonstrating that Derinat suppressed the UVB-induced DNA damage in a dose dependent manner in mouse skin. Taken together, our *in vivo* results revealed that Derinat effectively decreases the UVB-induced negative effects on skin, such as desquamation, epidermal proliferation, intracellular ROS production, DNA damage and the expression of COX-2 in BALB/c-nu mouse skin. Interestingly, the response of HDF to UVB irradiation was not as great as that of KCs based on the results presented here.

As shown in Figure 2, the intracellular Ca^2+^ elevation in KCs is seven times higher than that in HDF following UVB exposure. A similar tendency was also demonstrated in the ratio of ROS production and that of DNA damage in HDF. The differences between KCs and HDF may be attributed to differences in the intracellular Ca^2+^ elevation due to the different distribution of TRPCs between KCs and HDF for UVB-induced damage processes in the skin.

## 3. Experimental Section

### 3.1. Cell Culture

Human primary keratinocytes (KCs) and human dermal fibroblasts (HDF) were isolated from foreskins obtained via routine circumcision (with approval from the Institutional Review Board/Ethics Committee (IRB), number KMUH-IRB-960119), as described previously [43,44]. Briefly, the foreskin samples were washed using phosphate-buffered saline (PBS) (Gibco Invitrogen, Carlsbad, CA, USA) and disinfected with 70% alcohol. The harvested foreskins were minced into small pieces. To isolate the HDF, small pieces of the dermis were immersed in type I collagenase (3 mg/mL) (Sigma Chemicals Co.; St. Louis, MO, USA) and type IV collagenase (3 mg/mL) (Sigma Chemicals Co.) at 37 °C for 2 h. The samples were then incubated in 0.25% trypsin-EDTA solution (Gibco Invitrogen) at 37 °C for 30 min. The isolated HDF were grown in Dulbecco’s modified Eagle’s medium DMEM (Gibco Invitrogen) supplemented with 10% fetal bovine serum (FBS) at 37 °C in a humidified incubator containing a 5% CO_2_-in-air atmosphere. To isolate KCs, the small pieces of foreskin sample were treated with 1 U/mL dispase II (neutral protease) (Gibco Invitrogen) at 4 °C overnight. The epidermis was then separated from the foreskin sample and incubated in 0.25% trypsin-EDTA solution at 37 °C for 15 min. The isolated KCs were cultured in Keratinocyte-SFM medium (Gibco Invitrogen) at 37 °C in a humidified incubator containing a 5% CO_2_-in-air atmosphere. The medium was changed every 3 days.

### 3.2. Cell Viability Assay

Skin cells, 2 × 10^5^ KCs or 1 × 10^5^ HDF, were spread on 35-mm diameter dishes. Derinat (Technomedservia Pharmaceutical Company, Mironovskaya, Russia) was applied after the cells had spread on the dishes for 2 h, then incubated for 24 h at 37 °C in humidified 5% CO_2_. Skin cells were irradiated with 50 mJ/cm^2^ in KCs and 100 mJ/cm^2^ in HDF by UVB (UV source, 5 × 8-watt, 312 nm; BLX-312, Vilber Lourmat, France) and replacing normal medium for incubating 24 h. The ratios of the viabilities of skin cells were determined using 3-(4,5-dimethyl-2-thiazolyl)-2,5-diphenyl-2*H*-tetrazolium bromide (MTT) (Sigma Aldrich, St. Louis, MO, USA) at 570 nm in an ELISA reader [45].

### 3.3. Calcium Imaging

The intracellular Ca^2+^ responses were induced by applying thapsigargin (TG) (Sigma Aldrich), 1-oleoyl-2-acetyl-sn-glycerol (OAG) (Sigma Aldrich) and adenosine triphosphate (ATP) (Sigma Aldrich), according to previously described methods [44]. Before the experiments, cells were incubated with 1 μM Fluo-4-AM (Molecular Probes, Eugene, OR, USA) at 37 °C for 20 min and then washed with a balanced salt solution (BSS) buffer (5.4 mM KCl, 5.5 mM d-glucose, 1 mM MgSO_4_, 130 mM NaCl, 20 mM Hepes pH 7.4, and 2 mM CaCl_2_). Intracellular Ca^2+^ concentrations were calculated due to the ratio of fluorescence intensities emitted upon excitation with consecutive 3-s pulses of 488-nm light at a resolution of 1376 × 1038 pixels using an Olympus Cell^R IX81 fluorescence microscope (Olympus, Tokyo, Japan) equipped with an MT 20 illumination system (Olympus) and UPLanApo 10× objective lens. The intracellular Ca^2+^ concentration was measured based on calibration curves as follows. A Ca^2+^ calibration curve was created using a Ca^2+^ Calibration Buffer kit (Molecular Probes). Intracellular Ca^2+^ ([Ca^2+^]_i_) was estimated from Fluo-4 excited at 488 nm and imaged using an Olympus Cell^R IX81 fluorescence microscope and UPLanApo 10× objective lens at 20 °C. Fluo-4 signals were calibrated by measuring the fluorescence intensity from microcuvettes containing 10 mM K2-EGTA (pH 7.20) buffered to various [Ca^2+^] levels. Ca^2+^ concentration was analyzed using the following formula: [Ca^2+^]_i_ = KD × (F − Fmin/Fmax − F). Plotting the fluorescence intensity *versus* [Ca^2+^] yielded the calibration curve with the formula of: [Ca^2+^]_i_ = KD × (F − Fmin/Fmax − F), where KD = 345 nM, F = Fluo-4 intensity, Fmax = 640, and Fmin = 21.7 for Fluo-4.

### 3.4. Immunofluorescence Assay

The ratio of the DNA damage marker 8-oxodG was determined with an immunofluorescence assay using an antibody against 8-oxodG (Merck Millipore, Darmstadt, Germany). KCs and HDF were treated with or without Derinat and were cultured on 24 mm coverslips in 35 mm 6-well plates. After 24 h, cells were irradiated with 50 mJ/cm^2^ and 100 mJ/cm^2^ by UVB respectively, then replaced normal medium for incubating 24 h. After three washes with PBS, the cells were fixed by incubation with BD Cytofix for 10 min. The fixed cells were then briefly washed with PBS and incubated overnight at 4 °C in PBS containing 5% goat serum and 1% BSA with the appropriately diluted monoclonal antibody, 8-oxodG. After three washes with PBS, the cells were incubated for 1 h at room temperature with Alexa 488-conjugated goat anti-mouse IgM (Invitrogen) for 8-oxodG. The coverslips were washed three times with PBS (5 min each) and counterstained with 500 ng/mL 4,6-diamidino-2-phenylindole (DAPI, Sigma Aldrich) for 3 min. The coverslips were slide-mounted with antifade mounting solution and imaged using an Olympus FV1000 laser-scanning microscope (Olympus).

### 3.5. Analysis of Intracellular ROS Production in Skin Tissue

The animal experiments as affidavit of approval of animal use protocol Kaohsiung Medical Univerity, IACUC approval number: 101119. Briefly, we cooperated with SOMAPEX BIOTECH. CO. (Kaohsiung, Taiwan) to develop a special hydrogel for Derinat (Derinat hydrogel). Then male BALB/c-nu mice (6 weeks old) were purchased from the National Laboratory Animal Breeding and Research Center (Taipei, Taiwan). There were two mice in each group (three independent experiments) covered with Derinat hydrogel or pure hydrogel for 3 h and then irradiated with 360 mJ/cm^2^ UVB. After seven days treatment, a small section of skin tissue was excised from the dorsal area in BALB/c-nu mice and stained with 5 μM DHE (dye for staining intracellular ROS) for 30 min. After staining, the skin tissue was embedded in 1.5% low gelling agarose, sectioned into 100 μm slices with a DSK Microslicer (DTK-1000, TED PELLA, INC., Redding, CA, USA) and mounted on coated slides. The intracellular ROS production in skin tissue was observed with an Olympus FV1000 laser-scanning microscope.

### 3.6. Immunohistochemistry of Skin Tissue

Briefly, the dorsal skin from BALB/c-nu mice was fixed and embedded in paraffin. The monoclonal antibody of 8-oxo-2′-deoxyguanosine (8-oxodG) (Merck Millipore, 1:200) [46] and polyclonal antibody of cyclooxygenase (COX)-2 (Abcam, Cambridge, UK, 1:500) were used as previously described [6]. The immunoreactivity was visualized by incubation with DAB substrate-chromogen solution (DAKO) according to the manufacturer’s protocol.

### 3.7. Quantitative Reverse Transcription Polymerase Chain Reaction (qRT-PCR)

Total RNA was extracted from the dorsal skin of BALB/c-nu mice with the Trizol reagent (Invitrogen). Reverse-transcriptase reactions required 1 μg RNA to synthesize complementary cDNA using an RT kit (Invitrogen). Incubation conditions included 10 min at 25 °C, 120 min at 37 °C, and 5 min at 85 °C. The resulting cDNAs were utilized to detect TRPC7 expression level by the quantitative PCR using SybrGreen PCR Master Mix Kit (Applied Biosystems, Carlsbad, CA, USA) and specific primers: human TRPC1 (GenBank accession number, NM_003304), forward: TAG TGA CGA GCC TCT TGA CAA and reverse: CTG GCA GTT AGA CTG GGA GA; human TRPC4 (GenBank accession number, NM_003306), forward: CTC GCT GGT ACG ATG AGT TTC and reverse: GTG GGC TTT TGG GAG CTA TCA; human TRPC6 (GenBank accession number, NM_004621), forward: GTG ATC GCT CCA CAA GCC TAT and reverse: CTG CCA ACT GTA GGG CAT TCT; human TRPC7 (GenBank accession number, NM_001167576), forward: CGA GAA ACA GCG GAA AGA CTC and reverse: TCT GGC TAA CTC GTT GCT GAG; human GAPDH (GenBank accession number, NM_ 002046), forward: TGC ACC ACC AAC TGC TTA GC and reverse: GGC ATG GAC TGT GGT CAT GAG; mouse TRPC7 (GenBank accession number, NM_012035), forward: AAC CTG ACA GCC AAT AGC ACC TTC and reverse: TGG GCC TTC AGC ACG TAT CTC; mouse GAPDH (Gene Bank accession number, NM_001001303), forward: TGT GTC CGT CGT GGA TCT GA and reverse: TTG CTG TTG AAG TCG CAG GAG [47]. Thermal cycling was employed on the Applied Biosystems 7900HT fast real-time PCR system using the following cycling conditions: 95 °C for 10 min, and 40 cycles at 95 °C for 5 s, and 60°C for 30 s. Each complete amplification stage was followed by a dissociation stage at 95 °C for 15 s and 60 °C for 30 s.

### 3.8. Statistical Analysis

GraphPad Prism (La Jolla, CA, USA) was used to generate bar charts; error bars indicate standard deviations. A one-way, two-tailed analysis of variance (ANOVA) was also utilized to compare the means of each group. A *p-*value of less than 0.05 for differences between groups was considered statistically significant.

## 4. Conclusions

Our findings demonstrate that water soluble sodium deoxyribonucleate (Derinat), protects skin cells from UVB-induced damage by suppressing TRPCs-activated Ca^2+^ entry. The inhibition of intracellular Ca^2+^ elevation also contributed significantly to the decreased level of intracellular ROS production by mitochondria, which mitigated DNA damage in skin cells. The results of the *in vivo* experiments further supported that Derinat attenuated intracellular ROS production, COX-2 expression and DNA damage in the skin of BALB/c-nu mice exposed to UVB for seven days. Therefore, Derinat attenuates UVB-induced damage and has a great potential for the treatment of age-associated diseases or symptoms. This compound had no observable side effects, which makes it suitable for a myriad application in the fields of health and medicine.
